# Demographic disparities in the limited awareness of alcohol use as a breast cancer risk factor: empirical findings from a cross-sectional study of U.S. women

**DOI:** 10.1186/s12889-024-18565-z

**Published:** 2024-04-18

**Authors:** Monica H. Swahn, Priscilla Martinez, Adelaide Balenger, Justin Luningham, Gaurav Seth, Sofia Awan, Ritu Aneja

**Affiliations:** 1 Health Promotion and Physical Education, Wellstar College of Health and Human Services, Kennesaw State University, Kennesaw, GA USA; 2 Alcohol Research Group Public Health Institute, Emeryville, CA USA; 3 School of Public Health, Georgia State University, Atlanta, GA USA; 4 School of Public Health, University of North Texas Health Science Center, Fort Worth, TX USA; 5 School of Health Professions, University of Alabama at Birmingham, Birmingham, AL USA

**Keywords:** Breast cancer, Alcohol use, Awareness, US women

## Abstract

**Background:**

Alcohol use is an established yet modifiable risk factor for breast cancer. However, recent research indicates that the vast majority of U.S. women are unaware that alcohol use is a risk factor for breast cancer. There is limited information about the sociodemographic characteristics and alcohol use correlates of awareness of the alcohol use and breast cancer link, and this is critically important for health promotion and intervention efforts. In this study, we assessed prevalence of the awareness of alcohol use as a risk factor for breast cancer among U.S. women and examined sociodemographic and alcohol use correlates of awareness of this link.

**Methods:**

We conducted a 20-minute online cross-sectional survey, called the ABLE (Alcohol and Breast Cancer Link Awareness) survey, among U.S. women aged 18 years and older (*N* = 5,027) in the fall of 2021. Survey questions assessed awareness that alcohol use increases breast cancer risk (yes, no, don’t know/unsure); past-year alcohol use and harmful drinking via the Alcohol Use Disorders Identification Test (AUDIT); and family, health, and sociodemographic characteristics. We conducted multivariate multinomial regression analysis to identify correlates of awareness that alcohol use increases breast cancer risk.

**Results:**

Overall, 24.4% reported that alcohol use increased breast cancer risk, 40.2% reported they were unsure, and 35.4% reported that there was no link between alcohol use and breast cancer. In adjusted analysis, awareness of alcohol use as a breast cancer risk factor, compared to not being aware or unsure, was associated with being younger (18–25 years old), having a college degree, and having alcohol use disorder symptoms. Black women were less likely than white women to report awareness of the alcohol use and breast cancer link.

**Conclusions:**

Overall, only a quarter of U.S. women were aware that alcohol use increases breast cancer risk, although 40% expressed uncertainty. Differences in awareness by age, level of education, race and ethnicity and level of alcohol use offer opportunities for tailored prevention interventions, while the overall low level of awareness calls for widespread efforts to increase awareness of the breast cancer risk from alcohol use among U.S. women.

## Introduction

The U.S. Centers for Disease Control estimated there were just under 240,000 new female breast cancer cases in the U.S. in 2020, making it the most commonly diagnosed cancer among U.S. women [1]. Further, U.S. breast cancer cases have been steadily increasing over the last 2 decades, from an age-adjusted incidence-rate of 122.8 cases per 100,000 women in 2003, to 129.7 cases per 100,000 women in 2019. There is growing interest and a sense of urgency in identifying and targeting modifiable risk factors such as lifestyle behaviors to enhance prevention efforts for cancers overall, including breast cancer.

Alcohol use is an established and modifiable risk factor for breast cancer [2–7], with approximately 4–10% of breast cancer cases attributable to alcohol use each year, suggesting up to 26,400 breast cancer cases in 2019 were due to alcohol use [8]. The association between alcohol use and breast cancer is dose-dependent [5, 6], where heavy drinking is linked to the greatest increase in the risk of breast cancer, but light and moderate drinking also increase breast cancer risk. A meta-analysis by Bagnardi and colleagues [5] estimated a 1.04 relative risk for breast cancer among light drinkers compared with abstainers/occasional drinkers, 1.23 for moderate drinkers, and 1.61 for heavy drinkers [5].

Alcohol use as a breast cancer risk factor is of particular concern given how common and frequent any and heavy alcohol use are among women in the U.S. According to the most recent Behavioral Risk Factor Surveillance System (BRFSS) data, 46.6% of U.S. women consumed at least one drink in the last 30 days, and 11.3% of U.S. women engaged in binge drinking (four or more drinks in one sitting) [9]. Data from the most recent 2019–2020 National Alcohol Survey, which has been shown to produce alcohol use estimates more consistent with alcohol sales data than the BRFSS [10], show that 70% of women reported any past-year alcohol use and 24% reported any binge drinking (personal communication). Importantly, alcohol use among women in the U.S. has been increasing over the last several decades [11, 12]. In fact, Grucza and colleagues [12] found a significant increase in any alcohol use and binge drinking among U.S. women from 2000 to 2016. Moreover, recent research suggests increased alcohol consumption [13, 14] and higher alcohol use disorder-related mortality rates [15] during the COVID-19 pandemic, likely exacerbating the trends observed over the last several decades.

Even though 70% of U.S. women report past-year alcohol use, data are scarce on whether women know the risks of alcohol use specifically related to breast cancer. Khushalani and colleagues [16] found that only 24.6% of U.S. women aged 15–44 years knew that alcohol use is a risk factor for breast cancer, based on data from the nationally representative National Survey of Family Growth (data collected in 2011–2015) [16]. Among women who reported past 30-day alcohol use, 21% of those who did not report any binge drinking and 25.1% of those who did, were aware of this risk; among past 30-day abstainers, 26.6% were aware of this risk. The proportion of women who were aware of the breast cancer risks of alcohol use did not vary significantly by education, income, insurance, rurality, marital status, or family history of breast cancer [16]. However, women with a college education were less likely to indicate that they did not know or had no opinion on the link between alcohol and breast cancer rather than women with some or no college education. Also, non-Hispanic White women were less likely to endorse alcohol use as a breast cancer risk factor compared to Hispanic/Latinas and women from other non-Hispanic races [16].

Other studies in the U.S. have also shown similarly low awareness of the association between drinking and breast cancer. According to a survey of undergraduate and graduate students at a public university in the southeast, only 3% of participants indicated that alcohol use increased the risk of breast cancer [17]. In a sample of attendees of legal drinking age at the Minnesota State fair, 38% responded that alcohol use caused breast cancer [18]. Participants who reported past 30-day binge drinking were more likely than those who did not binge drink to indicate alcohol use as a risk factor for breast cancer, whereas awareness of this link was lower among Black/African American participants compared to other participants, although this was not statistically significant [18].

Collectively, the few studies conducted to date of the awareness among U.S. women about the link between alcohol use and breast cancer indicate a pervasive and low awareness and offer an important first step in understanding characteristics associated with awareness of the link between alcohol use and breast cancer. However, these studies are limited to particular age groups (women aged 15–44), specific geographic locations (the U.S. southeast and Minnesota), and to certain populations (undergraduate students). They also had limited inclusion of racial and ethnic minorities, making the observation of any statistically significant differences less feasible and robust. An understanding of the level and correlates of the awareness between alcohol use and breast cancer among a broad swath of women in the U.S. would provide a better understanding of the current state of awareness of alcohol use as a breast cancer risk factor. Additionally, to the best of our knowledge, only two studies (Calvert et al. and Kushalani et al.) have examined the associations between self-reported alcohol use and awareness of the breast cancer risk, and the alcohol use measure in both studies was limited to past 30-day use. Given that alcohol use may vary substantially over the year [19], employing a past 30-day alcohol use measure may limit the identification and inclusion of women who may drink heavily, but did not so in the past 30 days, and this group of women may be especially important for interventions given the higher risk of breast cancer at higher levels of alcohol use.

Therefore, this study aims to (1) assess the level of awareness of alcohol use as a risk factor for breast cancer in a large study of U.S. women and (2) to identify differences in awareness by sociodemographic characteristics and past-year level of alcohol use and alcohol-related problems, and (3) to examine the association between past-year level of alcohol use and problems and level of awareness that alcohol use increases breast cancer risk controlling for relevant covariates. Based on previous findings we hypothesized that awareness of alcohol use as a breast cancer risk factor will be low (∼ 30%), and that awareness will be higher among women who report heavy drinking and have a college education.

## Methods

### Participants and procedures

We collaborated with Qualtrics to distribute an online survey to 5,027 women between September 16 to October 14, 2021. We refer to this project as the Alcohol and Breast Cancer Link Awareness (ABLE) survey. All participants provided consent at the beginning of the survey. The study methodology has been described previously [20]. Eligibility criteria included residence in the U.S., ≥ 18 years of age, and identification as female. We provided the following quotas to Qualtrics to ensure a diverse sample and representation by age and race and ethnicity: 20% aged 18–24, 20% 25–34, 20% 35–44, 20% 45–54, and 20% aged ≥ 55; ≥15% Black/African American and ≥ 10% Spanish, Hispanic, or Latina. Qualtrics recruited participants using a network of collaborating national online panels which have volunteers who can choose to participate in any given survey. Because of this recruitment strategy, a response rate could not be computed. Women who participated in the survey received compensation at the discretion of Qualtrics. This study was approved by the Institutional Review Board of Georgia State University (H21673).

### Measures

Demographic measures included age, income, level of education, race and ethnicity, U.S. region of residence, and if they lived in a rural or urban setting. Other covariates included family history of breast cancer, source of medical advice, and self-reported health. To assess family history of breast cancer, participants were asked “Has anyone in your family ever had breast cancer?”, with response options of yes, no, and maybe/don’t know. We assessed participants’ preferred source of health information with the query “Imagine that you had a strong need to get information about health or medical topics. Where would you go first?”, with response options of website/online; social media (e.g., Instagram, Snapchat, Twitter); family; friend or co-worker; doctor or health care provider; books or library; magazines or newspapers; telephone information number, helpline, nurse advice line; complementary or alternative provider (e.g., acupuncturist, chiropractor, osteopath); and “other”. Participants were asked to mark one only [21]. We also assessed self-reported health with the question “In general, would you say that your health is excellent, very good, good, fair, or poor?”

We used the Alcohol Use Disorders Identification Test (AUDIT) to assess the level of alcohol use and problems over the past 12 months. We computed summary scores and constructed a categorical variable according to cutoff points recommended by the World Health Organization (WHO): abstainer = 0; low-risk consumption = 1–7; harmful use = ≥ 8; possible moderate-to-severe alcohol use disorder (AUD) = ≥ 15 [22, 23].

We assessed the dependent measure (belief alcohol use increases breast cancer risk) by asking participants “Do you think your risk of developing the following types of cancer is increased by drinking alcohol?”, and included eight different cancers (bladder, brain, breast, colon, liver, oral, ovarian, and stomach). Buykx and colleagues used this approach in their study assessing awareness of breast cancer risk from alcohol [24]. Participants could respond yes, alcohol consumption increases risk; no, alcohol consumption does not increase risk; or that they were unsure/don’t know. For context, of the eight cancer types asked, ovarian, brain, and bladder cancers are not associated with alcohol [24]. We constructed a 3-level variable indicating if the participant believed alcohol use increases the risk of breast cancer specifically with the categories yes, no, or don’t know. We did not include responses regarding the other cancers in our analyses.

### Analysis

In univariate analysis we produced frequencies to identify prevalence for the levels of awareness (yes, no, don’t know) that alcohol use increases the risk of breast cancer. In bivariate analysis, we used chi-square tests of independence to identify differences in the level of awareness of breast cancer risk from alcohol use by age, income, level of education, race and ethnicity, U.S. region, rural/urban status, family history of breast cancer, self-reported general health, source of health information, and AUDIT category. In multivariable analysis we used multinomial logistic regression to examine associations between response to the alcohol risk for breast cancer measure and sociodemographic characteristics (i.e., age, income, education, race and ethnicity, U.S. region, rural/urban status) and AUDIT category, controlling for relevant covariates (i.e., self-reported general health, family history of breast cancer, source of health information). All analyses were conducted in R version 4.2.2 [25]. Data management and descriptive data were aided by the dplyr package [26] and multinomial models were fit with the nnet package [27].

## Results

### Characteristics of participants

Due to small group sizes for other racial and ethnic groups, the current study was limited to only White non-Hispanic, Black non-Hispanic, and Hispanic/Latina participants, resulting in the omission of 257 respondents. Further, 53 individuals did not provide a response to the dependent measure of breast cancer risk associated with alcohol use. This resulted in a total sample of 4,717 individuals. Sample characteristics are presented in Table 1. The mean age of the 4,717 respondents was 41.2 years, and 23% had a bachelor’s degree or higher. Overall, 73%of the respondents were non-Hispanic White, 13.7% were Black, and 13.2% were Hispanic/Latina. 34% reported no alcohol use in the past year, 48.3% reported low-risk alcohol use, 10.3% reported hazardous alcohol use, and 7.5% reported use and problems consistent with having an AUD.

**Table 1:** Awareness of alcohol as a risk factor for breast cancer by key variables among U.S. adult women in the ABLE survey (*N* = 4,717)

	Knowledge alcohol use increases breast cancer risk			
	Do not know	No	Yes	*p*-value
	n (%)	n (%)	n (%)	
**Total**	1895 (40.2)	1669 (35.4)	1153 (24.4)	
**Age**				< 0.001
18–25	362 (35.0)	398 (38.5)	273 (26.4)	
26–35	333 (35.5)	381 (40.7)	223 (23.8)	
36–45	382 (40.2)	348 (36.6)	221 (23.3)	
45–55	402 (44.2)	304 (33.4)	204 (22.4)	
56+	416 (47.0)	238 (26.9)	232 (26.2)	
**Annual Income**				
$0 to <$25K	719 (39.8)	682 (37.8)	405 (22.4)	< 0.001
$25K to <$50K	517 (39.9)	465 (35.9)	315 (24.3)	
$50K+	495 (38.4)	419 (32.5)	374 (29.0)	
**Education**				< 0.001
Less than high school	127 (44.6)	108 (37.9)	50 (17.5)	
High school graduate	1347 (40.5)	1231 (37.0)	745 (22.4)	
College or more	412 (37.9)	323 (29.7)	353 (32.4)	
**Race/ethnicity**				0.041
Black Non-Hispanic	255 (39.4)	255 (39.4)	138 (21.3)	
Hispanic/Latina	231 (37.1)	233 (37.5)	158 (25.4)	
White Non-Hispanic	1409 (40.9)	1181 (34.3)	857 (24.9)	
**U.S. Region**				< 0.001
Midwest	454 (41.2)	388 (35.2)	261 (23.7)	
Northeast	259 (36.0)	242 (33.6)	219 (30.4)	
South	851 (40.4)	793 (37.7)	460 (21.9)	
West	330 (41.8)	246 (31.2)	213 (27.0)	
**Rural/urban**				0.168
Urban	1503 (40.5)	1288 (34.7)	920 (24.8)	
Rural	392 (39.0)	381 (37.9)	233 (23.2)	
**Self-Reported Health**				
Poor	103 (45.4)	73 (32.2)	51 (22.5)	
Fair	504 (47.2)	354 (33.1)	210 (19.7)	
Good	691 (39.9)	621 (35.8)	421 (24.3)	
Very Good	465 (38.2)	433 (35.6)	319 (26.2)	< 0.001
Excellent	129 (27.7)	185 (39.7)	152 (32.6)	
**Family history of breast cancer**				
Yes	726 (38.4)	599 (33.2)	478 (26.5)	
No	920 (48.6)	923 (38.2)	573 (23.7)	< 0.001
Maybe/Don’t know	246 (13.0)	140 (30.0)	97 (20.1)	
**Source of medical advice**				
Doctor/healthcare provider	835 (42.1)	679 (34.2)	471 (23.7)	
Website/online	672 (41.2)	544 (33.4)	414 (25.4)	
Family	171 (35.0)	201 (41.2)	116 (23.8)	< 0.001
Social media	73 (29.9)	108 (44.3)	63 (25.8)	
Other	141 (39.1)	133 (36.8)	87 (24.1)	
**AUDIT category**				
Abstainer	696 (43.5)	497 (31.0)	408 (25.5)	
Low-risk consumption	945 (41.5)	830 (36.4)	504 (22.1)	
Harmful consumption	153 (31.6)	214 (44.2)	117 (24.2)	< 0.001
Probable alcohol use disorder	101 (28.6)	128 (36.3)	124 (35.1)	

### Bivariate differences in awareness

Overall, 24.4% of the women identified alcohol use as a risk factor for breast cancer, 40.2% indicated that they were unsure, and 35.4% reported there was no link between breast cancer and alcohol use. In bivariate analyses there were statistically significant differences across the 3 levels of awareness by age, income, level of education, race and ethnicity, U.S. region of residence, family history of breast cancer, source of medical advice, and level of alcohol use and alcohol problems (see Table 1).

### Multinomial regression results

#### Yes vs. no

Table 2 provides the results from the multinomial regression model. Women with a college degree or higher (OR = 1.67, 95% CI (1.37, 2.04)) were more likely to indicate that alcohol use increases the risk of breast cancer than to respond it does not, compared to those with a high school education. Compared to White women, Black women were less likely (OR = 0.78, 95% CI (0.61,0.99)) to endorse alcohol use as a risk factor for breast cancer relative to responding it does not, and women with either past-year low-risk (OR = 0.68, 95% CI (0.57,0.82)) or hazardous alcohol use (OR = 0.69, 95% CI (0.52,0.91)) were similarly less likely to endorse alcohol use as a breast cancer risk factor compared to past-year abstainers.

**Table 2:** Multinomial model results comparing levels of awareness of alcohol increasing the risk of breast cancer among U.S. adult women in the ABLE survey

Awareness of alcohol as breast cancer risk factor		Yes vs. No	Yes vs. Don’t know	Don’t know vs. No
Variable	Category	OR (95% CI)	OR (95% CI)	OR (95% CI)
Age	18–25	ref	ref	ref
	26–35	**0.76 (0.60, 0.97)**	0.78 (0.61, 1.00)	0.98 (0.78, 1.22)
	36–45	0.88 (0.68, 1.12)	**0.75 (0.58, 0.96)**	1.17 (0.93, 1.46)
	45–55	0.94 (0.73, 1.22)	**0.75 (0.58, 0.97)**	1.25 (0.99, 1.58)
	56+	1.26 (0.96, 1.65)	**0.73 (0.56, 0.94)**	**1.74 (1.36, 2.22)**
Income	$0 to <$25K	ref	ref	ref
	$25K to <$50K	1.08 (0.88, 1.31)	1.00 (0.82, 1.21)	1.08 (0.91, 1.28)
	$50K+	1.17 (0.95, 1.45)	1.06 (0.86, 1.30)	1.11 (0.92, 1.34)
Education	Did not graduate High School	0.75 (0.51, 1.09)	0.78 (0.53, 1.13)	0.96 (0.72, 1.29)
	Graduated High School	ref	ref	ref
	Bachelor’s degree or higher	**1.67 (1.37, 2.04)**	**1.50 (1.24, 1.82)**	1.11 (0.92, 1.34)
Race and Ethnicity	White non-Hispanic	ref	ref	ref
	Black/African American, non-Hispanic	**0.78 (0.61, 0.99)**	0.97 (0.76, 1.24)	**0.80 (0.65, 0.99)**
	Hispanic/Latinx	0.93 (0.73, 1.18)	1.02 (0.81, 1.30)	0.90 (0.73, 1.12)
AUDIT	Abstainer	ref	ref	ref
	Low-risk Consumption	**0.68 (0.57, 0.82)**	**0.78 (0.66, 0.93)**	0.87 (0.74, 1.02)
	Hazardous Consumption	**0.69 (0.52, 0.91)**	1.11 (0.83, 1.48)	**0.62 (0.48, 0.80)**
	Probable alcohol use disorder	1.23 (0.91, 1.67)	**1.85 (1.34, 2.55)**	**0.67 (0.49, 0.91)**

*Note* Model controlled for US region of residence, urban/rural status, self-reported health, source of medical info. Bolded text indicate significance at the *p* < 0.05 level

#### Yes vs. don’t know

All age groups above 35 years of age were less likely to indicate alcohol use increases the risk of breast cancer than to respond they “don’t know” compared to women aged 18–25. Women with a college degree or higher (OR = 1.44, 95% CI (1.19,1.73)) compared with women with a high school education were more likely to indicate awareness rather than respond “don’t know”. Women who reported low-risk alcohol use (OR = 0.77, 95% CI (0.65,0.92)) were less likely to endorse alcohol use as a risk factor for breast cancer than to say they “don’t know”, compared to past-year abstainers. Women with probable AUD (OR = 1.85, 95% CI (1.34,2.55)) were more likely to indicate alcohol use increases the risk for breast cancer rather than state they “don’t know. There were no statistically significant differences by race and ethnicity.

#### Don’t know vs. no

Women above the age of 45 were more likely to say they “don’t know” alcohol use increases the risk of breast rather than saying it does not compared to women aged 18–25. Black women (OR = 0.80, 95% CI (0.65,0.98)) compared to White women were less likely to respond that they “don’t know” if alcohol use increases the risk of breast cancer than to respond it does not, as were women who reported either hazardous alcohol use (OR = 0.65, 95% CI (0.51, 0.83)) or had a probable AUD (OR = 0.66, 95% CI (0.49,0.89)), compared to past-year abstainers. There were no statistically significant differences by level of education.

## Discussion

This study aimed to assess the level of awareness of alcohol use as a risk factor for breast cancer among a large cross-section of U.S. adult women and to examine demographic differences and alcohol use and problems as correlates of awareness. Consistent with our hypothesis, our findings show low awareness that alcohol use increases breast cancer risk. Only about one in four women (24.4%) acknowledged that alcohol use increased their risk of developing breast cancer, while 40.2% indicated that they didn’t know or were unsure, and 35.4% reported alcohol use did not increase breast cancer risk. The proportion aware that alcohol use increases the risk of breast cancer is consistent with previous findings from national U.S. samples, which ranged from 29.9% among a national web panel sample of women in 2008 [28] to 25% in a representative sample of U.S. women collected between 2011 and 2015 (Khushalani et al., 2020). The level of awareness among U.S. women about the risk of breast cancer due to alcohol use seems to have remained fairly consistent over the first couple decades of the 21st century, which is concerning as alcohol use among U.S. adult women has been increasing over that same period [29]. An important contribution of this study is the observation that 40% of respondents reported either not knowing or being unsure if alcohol use increases the risk of breast cancer, which suggests an important opportunity for strategic health promotion messaging to convey the carcinogenic risk of alcohol use for women.

This study identified differences across several demographic characteristics in adjusted analyses in the awareness that alcohol use increases breast cancer risk. We observed that young women between the ages of 18–25 were more likely than their older counterparts to be aware of the breast cancer risk from alcohol use. This finding was consistent across comparisons between endorsement of awareness and both outright rejection and being uncertain. This heightened awareness among young women could potentially be attributed to or correlated with declines in youth drinking and a growing interest in adopting healthy lifestyle behaviors [30]. There is a notable increase in alcohol consumption among middle-aged to older women over the early 21st century [29], making the relatively lower awareness particularly concerning, and highlighting the need for targeted outreach to middle-aged and older women to increase understanding of breast cancer risks associated with alcohol use.

In adjusted analyses we also observed differences in a common measure of socioeconomic status, that is, level of education, but not in another common measure, namely, income. Specifically, we observed that women with a college degree or higher were more likely to be aware of the association between alcohol use and breast cancer risk compared to those with a high school education. These findings partially differ from those reported byJS Khushalani, J Qin, DU Ekwueme and A White [16], who found that awareness of increasing breast cancer risk from alcohol use among U.S. women did not differ based on education or income. In contrast, research conducted outside the U.S. has shown that awareness is generally higher among women with higher education levels [31, 32], and the literature generally supports a positive association between having more education and health knowledge [33]. Importantly, despite our finding that women who graduated from college were more often aware that alcohol use increased breast cancer risk, awareness within this group was still low in absolute terms, with only 30.7% of survey participants acknowledging this risk.

Our study revealed that Black women were less likely than non-Hispanic White women to recognize alcohol use as a risk factor for breast cancer, and they were also less likely to respond with uncertainty about this link. A similar study reported lower awareness among Black/African American participants compared to other participants, although this difference was not statistically significant [18]. However, the Calvert study also examined awareness of alcohol as a risk factor for *any* cancer and observed that Black people compared to White people were 24% less likely to be report awareness of this link, and this was statistically significant [18]. In contrast, Khushalani and colleagues [16], found that non-Hispanic White women were less likely to acknowledge alcohol use as a breast cancer risk factor compared to Hispanic/Latinas and women from other non-Hispanic racial backgrounds. Methodological differences including sampling, sample size and interviewing modality likely explain the differences in our findings compared to other studies. Our findings are consistent with existing racial and ethnic disparities in access to health information [34], and health literacy [33] and underscore the need for tailored messaging campaigns to reduce these disparities and ensure equitable distribution of health knowledge.

The observation that women who self-reported low-risk or hazardous alcohol use were less likely to acknowledge alcohol use as a breast cancer risk factor relative to abstainers’ contrasts findings from prior literature. Khushalani and colleagues reported that awareness was highest among women who reported no past-30-day alcohol use at 26.6%, although a similar prevalence of 25.1% was observed among women who reported past-30-day binge drinking [16]. On the other hand, women who self-reported probable alcohol use disorder were more likely to be aware of this link and less likely to respond that they did not know or were unsure about it. These latter findings are consistent with findings from Calvert and colleagues [18], that observed that participants who reported past-30-day binge drinking were more likely than those who did not binge drink to acknowledge alcohol use as a risk factor for breast cancer. Internationally, the literature is also mixed with some studies showing higher awareness among women who report recent alcohol use compared to those with no recent alcohol use [35], and others showing no association between average number of drinks consumed in the past 30 days and awareness that alcohol use increases breast cancer risk [36]. While we did not specifically examine this issue, it is possible that women engaging in low-risk or harmful consumption may rationalize their alcohol use as comparatively less risky relative to smoking or other substance use [28, 37].

Our finding that women who are experiencing AUD symptoms are more likely to be aware of the breast cancer risk from alcohol use may be the result of having sought treatment or information about reducing alcohol use. Continuing to use alcohol even after it causes physical or mental health problems is a key feature of AUD, suggesting awareness of alcohol’s carcinogenic effects may not be sufficient to deter use among women experiencing AUD symptoms. Future research should explore if and how alcohol’s health risks specific to women could be leveraged to support alcohol abstinence or reduction to mitigate alcohol-related health harms among women with AUD symptoms who are at especially high risk of negative health outcomes.

Communicating to women that alcohol use is a risk factor for breast cancer can inform their decisions about whether to use alcohol and, if they choose to do so, how much to consume. It is noteworthy that most women in our study already understood the link between alcohol and liver cancer (66%), which highlights that many women already recognize, to some extent, the carcinogenic effects of alcohol use. However, this awareness has yet to extend to knowledge about the link between alcohol use and other cancer types. Additionally, recent work suggests that awareness of the carcinogenic nature of alcohol is associated with support for alcohol control policies [38–41], which are known to reduce per capita alcohol consumption and population-level alcohol related harms [42]. Thus, increasing awareness of the carcinogenic effects of alcohol may increase public support for traditionally unpopular alcohol control policies such as taxation and make their enactment more likely.

### Limitations

Several important limitations should be considered when interpreting our findings. First, the Qualtrics-recruited sample was subject to the limitations inherent in conducting research using proprietary web panels. As Qualtrics used a broad recruitment strategy for survey distribution, we could not compute a response rate or compare characteristics between responders and non-responders in this cross-sectional survey. Second, because the consent form told women that the survey was about “*women’s knowledge and beliefs about alcohol and breast cancer*” and because the survey contained questions about alcohol use and breast cancer, the survey questions may have primed respondents to think about the link between alcohol use and breast cancer. Third, the survey was relatively brief, limiting the contextual factors that were collected and analyzed. More specifically, the AUDIT only addresses the frequency of alcohol and associated harms, not the specific types of alcohol consumed or other contextual factors regarding the alcohol use patterns or preferences. Fourth, our measure of alcohol use and problems was limited to a past-year timeframe, and we did not assess lifetime measures of alcohol use which may impact awareness of the breast cancer risk from alcohol use. Additionally, we did not include information about the types of alcohol consumed and whether women who consume hard liquor compared to wine or beer, as an example, would be more likely to recognize the risk associated with alcohol. Finally, because the survey was cross-sectional, causation could not be determined for any of the factors examined. Although these limitations are important, previous research has recognized the value of online surveys in other populations because of their ability to quickly gather epidemiological data [43, 44]. Moreover, our sample was relatively large, permitting robust samples sizes of demographic subgroups, such as racial and ethnic subgroups, not available in previous studies.

## Conclusion

This study provides valuable insights for understanding the limited awareness about the link between alcohol use and breast cancer risk in a large sample of U.S. women. Given that the overall prevalence of awareness was rather low at approximately 25%, efforts to inform the entire population of U.S. women of the breast cancer risk from alcohol use are warranted. However, the even lower levels of awareness observed among Black women and women with lower levels of education calls for targeted and tailored interventions to ensure equitable distribution of health knowledge. Importantly, about 40% of respondents expressed uncertainty about the link between alcohol use and breast cancer, which represent an opportunity to clarify any confusion or suspicions women may have about the negative health effects of alcohol use specific to women’s health. Future research should develop and test specific strategies to raise awareness about alcohol use as a modifiable risk factor for breast cancer applying non-judgmental, accessible, and informative approaches for women. Moreover, increasing awareness of specific alcohol harms to women can potentially lead to greater support for evidence-based alcohol control policies, which can have a significant impact on reducing alcohol-related harm in a population. Addressing the low awareness and misconceptions surrounding alcohol’s carcinogenic effects is crucial to address the rising incidence of breast cancer and reduce the negative health impacts of alcohol use.

## Data Availability

The ABLE survey dataset analyzed in this study is available from the corresponding author upon reasonable request.
